# “Palliative Pandemic Plan,” Triage and Symptoms Algorithm as a Strategy to Decrease Providers’ Exposure, While Trying to Increase Teams Availability and Guidance for Goals of Care (GOC) and Symptoms Control

**DOI:** 10.1177/1049909120942494

**Published:** 2020-07-21

**Authors:** Santiago Lopez, Gene Decastro, Katlynn M. Van Ogtrop, Sindee Weiss-Domis, Samuel R. Anandan, Christopher J. Magalee, Regina Roofeh, Tara A. Liberman

**Affiliations:** 1 North Shore University Hospital, Northwell Health, Manhasset, New York, NY, USA; 2 Long Island Jewish Medical Center, Northwell Health, New Hyde Park, New York, NY, USA

**Keywords:** COVID-19, novel coronavirus, palliative medicine, triage and symptoms algorithms

## Abstract

As the spread of the novel coronavirus disease 2019 (COVID-19) continues worldwide, health care systems are facing increased demand with concurrent health care provider shortages. This increase in patient demand and potential for provider shortages is particularly apparent for palliative medicine, where there are already shortages in the provision of this care. In response to the developing pandemic, our Geriatrics and Palliative (GAP) Medicine team formulated a 2-team approach which includes triage algorithms for palliative consults as well as acute symptomatic management for both patients diagnosed with or under investigation (PUI) for COVID-19. These algorithms provided a delineated set of guidelines to triage patients in need of palliative services and included provisions for acute symptoms management and the protection of both the patient care team and the families of patients with COVID-19. These guidelines helped with streamlining care in times of crisis, providing care to those in need, supporting frontline staff with primary-level palliative care, and minimizing the GAP team’s risk of infection and burnout during the rapidly changing pandemic response.

## Background

The first case of the 2019 novel coronavirus disease (COVID-19) was diagnosed in Wuhan, China, in early December 2019.^1^ By March 2020, the World Health Organization declared the COVID-19 spread as a pandemic.^2^ Older adults and those with comorbidities, particularly cardiovascular disease, diabetes, and chronic respiratory disease, are at highest risk for severe symptom burden and mortality.^1,3,4^ Intensive care unit admissions for patients with this disease varies from 5% to more than 20%, numbers that have shown to surpass the capabilities of even well-established health care systems.^4,5^ Data from China and Italy reported case mortality rates of up to 12.5% for those aged 70 to 79, 19.7% for those 80 to 89, and 22.7% for those older than 90.^4,6^ While this pandemic was evolving in China and Italy, it was known that a significant portion of the US population was in the high-risk mortality group, including the 13% aged 65 years and older, 51.7% with at least 1 chronic condition, and 31.5% with multiple chronic comorbidities.^7,8^ As with all patients with advanced illness, patients with or under investigation (PUI) for COVID-19 require complex symptom management, delicate Goals of Care (GOC) discussions, and psychosocial support for patients, families, caregivers, and health care workers. Although a palliative care approach is fundamental for this level of care, there are not enough palliative care physicians to meet the needs of these patients.^9^ As of March 18, 2020, in the United States, there were 7038 cases and up to 97 deaths. However, in less than 36 days those numbers escalated to 865 585 cases and 48 816 deaths.^10^ In early March 2020, understanding the foreseeable magnitude of the pandemic in the United States and the anticipated surge in demand for specialized level palliative care, the Geriatrics and Palliative (GAP) team at North Shore University Hospital developed a fundamental plan to address the projected palliative care need of the patients affected by COVID-19.^11^ Here, we describe a 2-team approach including triage algorithms for palliative consults and acute symptoms management for patients both diagnosed with and PUI for COVID-19. Furthermore, we provide a comparison with our pre-COVID-19 operational metrics to demonstrate the capabilities of this simple approach, triage tool, and treatment algorithm that can support efficiency and safety during times of crisis.

## Methods

In early March 2020, based on the literature and considering the need for a “palliative pandemic plan” the GAP Consult Service at a 738-bed quaternary care teaching center in New York developed algorithms to triage and manage symptoms, including end of life (EOL), for patients requiring a palliative care consult during times of surge demand. Using both external and health system resources, the algorithms were designed to prioritize patient needs while also minimizing physician risk of exposure to COVID-19.^12^ In order to achieve consensus of expertise and buy in from stake holders, an adapted Delphi method was utilized.^13^ As a result, these algorithms were developed through a dedicated literature review and a multidisciplinary partnership, including representation from physician, advanced care practitioner, social work, chaplaincy, and medical subspecialties.^14-17^ These multidisciplinary perspectives allow for an agile, unified response to rapidly shifting health care priorities in times of crisis.

**Figure 1.**
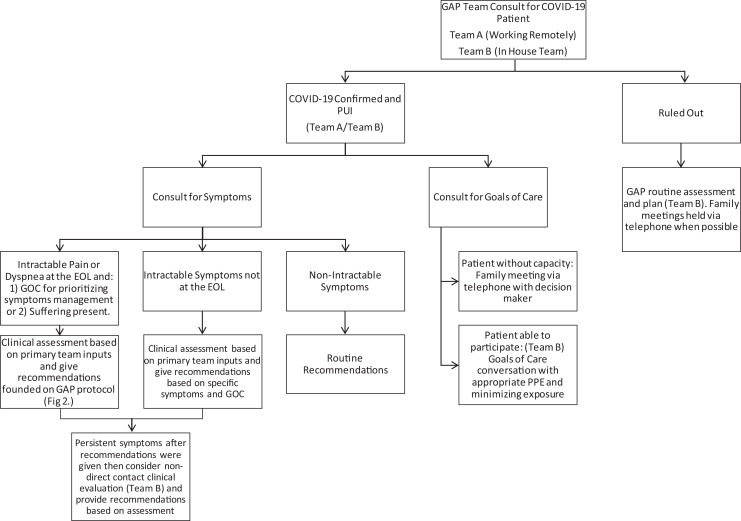
Algorithm for palliative care consult of patient with coronavirus disease 2019 (COVID-19) confirmed or patient under investigation (PUI) for COVID-19.

A vital step to appropriate treatment for patients during surge demand is the preservation of the GAP consult team: in our case 5 board-certified palliative care specialists, 2 full-time equivalent advanced care providers, a dedicated chaplain, and a social worker. A major risk of treating patients with COVID-19 is provider exposure and subsequent need for quarantine. This is a particular hazard for a relatively small consult service where the side-lining of any one individual causes a major reduction in the team’s overall ability to provide care. To minimize the risk of exposure to COVID-19, we developed a 2-team-based approach (Figure 1). Team A was working remotely and included 5 providers. This team managed complicated GOC discussions for those patients who were not able to communicate or did not have capacity. Additionally, they directed primary teams on addressing acute symptomatic management as well as uncomplicated GOC discussion. Team B, the in-house team, included 2 providers and oversaw giving recommendations on patients with persistent symptoms that were not able to be addressed by team A as well as addressing GOC for those patients with medical decision-making capacity and not able to be contacted by a mobile application or phone call. In order to achieve our work, the GAP team relied on mobile applications (Microsoft Teams, Doximity Dialer, and regular phone calls) for internal team meetings, GOC discussions with patients and families, and advising on primary-level palliative care for frontline providers. Precautions against provider burnout were also implemented, including flexible work hours, schedule rotation, and availability of additional support services.

**Figure 2.**
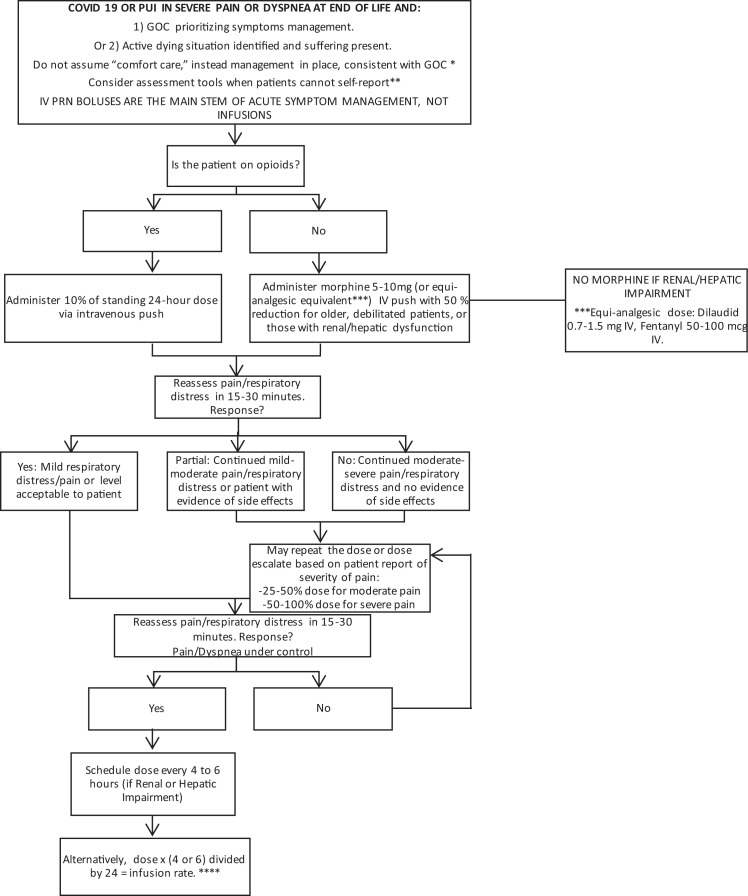
Algorithm for acute management of coronavirus disease 2019 (COVID-19) or patients under investigation (PUI) for COVID-19 in severe pain or dyspnea at the end of life (EOL). * Comfort care is a non-specific term that does not define a treatment plan. Therefore, specific treatments such as intravenous fluids, antimicrobials, and other therapies should be continued unless otherwise define by goals of care (GOC). ** Pain Assessment in Advanced Dementia Scale (PAIN AD) and Respiratory Distress Observation Scale (RDOS). *** Opioid equi-analgesic table as per individual institutional consensus. **** In patients with normal renal or hepatic function, adjusting the continues infusion at 8-12 hours (about five half-lives) is generally acceptable, as the drug will be close to or at steady state. If there is organ impairment, it is reasonable to wait up to 24 hours. PUI= Patient under investigation.

Understanding the high mortality associated with COVID-19, we developed a specific algorithm for the management of severe pain and dyspnea at EOL. This specific algorithm (Figure 2) provided recommendations for symptom treatment when GOC indicated prioritizing symptoms, or for actively dying situations where suffering was identified. The algorithm provides a stepwise approach for opioid selection, starting doses, titration, and scheduling around-the-clock regimens for pain or dyspnea management during EOL care. This tool was created not only to provide a framework for the GAP team but as a tool for frontline staff to address distressful symptoms at the EOL.

## Results

We developed an algorithm to streamline triage (Figure 1) and advanced symptomatic treatment (Figure 2) of patients with confirmed COVID-19 and PUI for COVID-19 diagnosis. The algorithms describe our 2 teams and emphasize the importance of appropriate symptomatic treatment, particularly for those in severe distress or actively dying. The expectation remained that a healthcare provider will evaluate the patient at bedside for acute respiratory distress and pain crisis, and address concerns of the patient, families, and staff. Treatment of pain crises and acute respiratory distress remains consistent with best known practices and expert’s recommendations.^14-17^

## Discussion

During the last decades, public health efforts and medical advances have promoted longevity and enabled the population to live longer and with multiple comorbid conditions. It is these vulnerable populations that COVID-19 is particularly affecting and overwhelming health care organizations worldwide. In the setting of this pandemic, many healthcare providers will have to triage patient needs to continue to provide appropriate, goal-concordant care. The use of predetermined algorithms is a helpful tool to guide triage of patient care during a time of crisis. When implemented appropriately, these algorithms can guide medical decision-making and acute symptomatic management to ensure patients are treated fairly and continue to be given high quality health care.

Prior to this pandemic, the average number of initial consults seen by the GAP team was 202 consults/month. Time from admission to consult and consult to discharge were 6.5 and 11.1 days respectively, length of stay (LOS) was 18 days, mortality rate was 38%, and 4% of our patients were discharged to hospice. However, during this pandemic, between March 23 and April 23, the number of patients seen by the GAP team with confirmed COVID-19 and PUI was 305. This number does not account for patients without a diagnosis of COVID-19 infection or PUI, therefore the actual number of consult seen was above the indicated number; however, due to a delay on data reporting by our general dashboard we cannot indicate the actual total number of consults at time of writing. Time from admission to consult and consult to discharge were 6.9 and 7 days respectively, LOS was 13.6 days, mortality rate was 69.5%, and 8.5% of these patients were discharged to hospice (Table 1).

**Table 1.** Baseline Characteristics of Operational Metrics for GAP Consult Team Prior to COVID-19 Versus March 23 and April 23, 2020 (Peak of COVID-19 in New York).

Operational metric	Average no. prior to COVID-19	Average no. March 23-April 23, 2020
No. consult per month	202	305
Admission to consult	6.5	6.9
Consult to discharge	11.1	7
LOS^a^	18	13.6
All mortality, (%)	38	69.5
Discharge to hospice, (%)	4	8.5

Abbreviations: COVID-19, coronavirus disease 2019; GAP, geriatrics and palliative; LOS, length of stay.

^a^Length of stay begins with admission time and ends with discharge time, time at death, or midnight on the last day of data collection for the study. It does not include time in the emergency department.

In our experience, these triage and symptomatic management algorithms allowed the GAP team to increase its consultation capability by 50% while maintaining the average time from admission to consult and decreasing the time from consult to discharge (11.1 days pre-COVID 19 vs 7 days during COVID-19). Additionally, the GAP team was able to support GOC discussion and advised with symptom management for a population with a high mortality (69.5% of the confirmed COVID-19 and PUI seen by the palliative care team died during the reported time). Furthermore, referrals to inpatient hospice increased from 4% to 8.5%. Finally, the algorithms above allowed for preservation of the GAP team. Up to May 5, 2020, there were no providers positive for COVID 19.

Follow-up studies can look into the replicability of these inpatient palliative care team triage and symptomatic management algorithms. Furthermore, it will be important to see if similar triage and symptoms management tools can be created for outpatient palliative care groups.

## Conclusion

In this article, we described a strategy we employed to proactively prepare for an exponential growth of patients with COVID-19 in New York. Our 2-team based approach, triage and symptoms algorithm allowed the GAP team to provide specialized palliative care while advising frontline staff during the peak of the COVID-19 pandemic. Our strategy also allowed for the preservation of our team, both from the infectious and burn out points of view. However, understanding the surge demand for specialized palliative care we have seen during this outbreak and the almost 80% increased on our baseline mortality (38% pre-COVID 19 vs 69.5% during COVID-19) it is important that health care system and health care officials proactively work on better allocating resources for inpatient palliative care teams so providing care during such difficult times remains sustainable.

Understanding that this is an unexpended and evolving situation, we recognized our protective strategies, triage system, and algorithm for symptoms management will need to be revised and adjusted as this pandemic develops. Hopefully, other strategies such as improving testing, creating dedicated COVID-19 facilities, improving medical equipment availability (eg, mechanical ventilators, venous-venous extracorporeal membrane oxygenation [ECMO])), and containment measures will be able to change the trajectory of this pandemic so proportionality, duty to provide care, reciprocity, equity, and trust can be appropriately applied.
